# Improving Access to Information and Support for Patients With Less Common Cancers: Hematologic Cancer Patients’ Views About Web-Based Approaches

**DOI:** 10.2196/jmir.1894

**Published:** 2011-12-21

**Authors:** Christine Louise Paul, Mariko Leanne Carey, Alix Edna Hall, Marita Clare Lynagh, Robert W Sanson-Fisher, Frans Alexander Henskens

**Affiliations:** ^1^Health Behaviour Research Group, Priority Research Centre for Health Behaviour, Hunter Medical Research InstituteSchool of Medicine and Public HealthUniversity of NewcastleCallaghanAustralia

**Keywords:** Cancer, hematologic diseases, information-seeking behavior, social support

## Abstract

**Background:**

Meeting the psychosocial needs of vulnerable groups such as cancer survivors remains an ongoing challenge. This is particularly so for those who have less access to the usual forms of medical specialist and in-person support networks. Internet-based approaches offer an opportunity to better meet patients’ information and support needs by overcoming the barrier of geographic isolation.

**Objective:**

The aim of the study was to assess the reported level of access to the Internet, preferred sources of information, and preferred sources of support among survivors of hematologic cancers.

**Method:**

A population-based, Australian state cancer registry invited eligible survivors to complete a survey about psychosocial needs, including items measuring Internet access and patterns of use. Of the 732 eligible survivors invited to participate, 268 (36.6%) completed and returned the pen-and-paper-based survey.

**Results:**

The majority of participants (186/254, 73.2%) reported a high level of access to the Internet, with higher Internet access associated with a higher level of education, larger household, younger age, and being married or employed. A total of 62.2% (156/251) of survivors indicated they were likely to use the Internet for accessing information, with the percentage much lower (69/251, 28%) for accessing support via the Internet. Likelihood of using the Internet for support was associated with feeling anxious and being employed.

**Conclusions:**

While the Internet appears to offer promise in increasing equitable access to information and support for cancer survivors for both metropolitan and regional areas, it is viewed less favorably for support and by particular subgroups (eg, older people and those without a university degree) within the survivor population. Promoting greater understanding of this mode of support may be required to achieve its potential. Information and support options other than Web-based approaches may continue to be needed by vulnerable groups of cancer survivors.

## Introduction

Although hematologic cancers such as lymphoma and leukemia are much less prevalent than other cancer types such as colorectal cancer or breast cancer [1], in developed countries such as Australia they are a major cause of cancer death, due to poor survival rates compared with other cancers [1]. Diagnosis and treatment can have a devastating impact on life expectancy, fertility and sexuality [2,3], and overall health [4]. Accordingly, these patients report a need for information [5] and support [6]. Canadian data indicate that rates of clinical distress among those with hematologic cancers can range from 32% to 48% given the debilitating nature of the disease and its treatment [7]. Australian data indicate that hematologic cancer patients are also often isolated from support systems due to the need to travel to major hospitals for treatment, with treatment potentially lasting several months [5,8].

The prevalence and nature of the disease and its treatment raise some particular issues for the provision of psychosocial support. The opportunities for peer support, for example, are limited by the relative rarity of an age- or gender-matched survivor being available for either face-to-face or telephone-based peer-support programs. The concentration of hematologic professionals in major centers also can result in low access to face-to-face information and support once a patient has completed a round of treatment, particularly for those who live in nonmetropolitan locations. Further, opportunities for social and peer support may be limited due to lengthy inpatient stays and restriction of activities due to risk of neutropenic infection. Therefore, it is likely that a suite of options needs to be made available in order that hematologic cancer patients receive sufficient information and support throughout the months and years that may follow diagnosis.

Alongside the vital role of specialist medical staff, the Internet offers unique advantages for the delivery of information and psychosocial support to hematologic cancer patients, primarily due to its high level of accessibility. Up to 77% of Australian cancer patients access information about cancer via the Internet [9]. Internet access in Australia has quadrupled between 1998 and 2008 [10]. The most recent Australian data suggest that 72% of the population have home Internet access [10], while in the United States up to 69% of people may have home Internet access [11]. For those in regional and remote areas [12] the Internet may overcome some geographic barriers. It provides a way of connecting with information, services, and others in a similar situation no matter their location or level of wellness. It also offers the opportunity to provide peer online forums to obtain support from others in similar positions, who may not be accessible face-to-face.

A small group of studies have explored the effectiveness of Web-based psychosocial support for cancer survivors using robust randomized controlled designs [13-17], with mixed findings for psychosocial outcomes. The single study that included hematologic cancer survivors [15] also involved participants with other types of cancer diagnoses and suggested that those who were single, older, and less educated were less likely to use the Web-based intervention. Issues of reach and access in relation to Web-based interventions have also rarely been addressed.

While Internet accessibility is apparently high and increasing, there are no current data about the accessibility of this resource for hematologic cancer patients. Internet access can differ according to income, education, age, and geographic location [12,18]. These differences may in turn create or exacerbate inequality. Given that the sociodemographic profile of adult hematologic cancer patients includes a substantial proportion of older age groups [1], it is important to establish whether older or disadvantaged patients have ready access to the Internet in a manner that is conducive to its use for obtaining support and information.

The study aimed to do the following in a cross-sectional sample of people with a diagnosis of a hematologic cancer: (1) investigate the proportion of metropolitan versus regional survivors who reported a high level of access to the Internet, (2) measure the proportion who reported being likely to use various sources (Internet, print, telephone, face-to-face) for information and support and the perceived benefits of Internet options, and (3) explore the sociodemographic characteristics of survivors who reported both a high level of Internet access and being likely to use the Internet for information or support.

## Methods

### Design

#### Sample

Through a population-based cancer registry we recruited survivors aged 18 to 80 years at study invitation who had a diagnosis of leukemia, lymphoma, or myeloma in the prior 3 years. Use of this registry permitted sampling across the full range of cancer types, locations, and stages of treatment.

#### Procedure

On behalf of the researchers the cancer registry sent all eligible patients a questionnaire package containing an invitation letter, information statement, prepaid envelope, registry brochure, self-report pen-and-paper survey, and questionnaire package for their principal support person. Patients who did not respond to the initial questionnaire after 4 weeks were mailed a reminder letter from the cancer registry and a second questionnaire package.

### Measure

The 30-minute self-report pen-and-paper survey comprised a series of measures regarding psychosocial issues for cancer survivors, a subset of which are reported here. Participants were asked about their use of the Internet, accessibility of the Internet, likelihood of using each of a range of options for seeking support or assistance, and perceived benefits and disadvantages of the Internet for cancer-related information and support. Multimedia Appendix 1 contains the Internet-related survey items. The Depression Anxiety Stress Scales [19], a reliable and valid measure for assessing psychological status in cancer patients [20], was also completed as part of the survey. Diagnosis, gender, age, and postcode (to assess metropolitan status) were obtained from registry records with the patient’s permission.

### Analysis

#### Metropolitan Versus Regional Categorization

Survivors’ residential postcodes were used to classify their location on the Accessibility/Remoteness Index of Australia (ARIA+) classification. Metropolitan was defined as the ARIA+ category major cities, and regional was defined as inner regional, outer regional, remote, or very remote.

#### Level of Internet Access

We reported proportions to describe level of Internet access on each access item. Chi-squares were used to compare metropolitan versus regional access on each item and on overall access score. An access score was calculated as follows. A high score consisted of 5 or more of the following responses: frequency of access (any/most of the time), connection problems (none/minor), privacy (moderately/very), comfort (very/moderately), printing (any/limited), and confidence (very/moderately). A moderate score was any 3 or 4 of these responses, and low was classed as a score of 0–2. A score of 0 was given to those who indicated they had no access to the Internet for personal use.

#### Likelihood of Using Various Modes of Information and Support

Response categories of likely and very likely were combined. Proportions and 95% confidence intervals were used to describe the data for each item.

#### Sociodemographic Characteristics Associated With Reported Internet Access and Likelihood of Using the Internet

We conducted initial chi-square analyses with the following independent variables: gender, living in a rural area, education, marital status, employment status, household size, health status, and whether the survivor had normal or some level of anxiety or depression. Age at diagnosis in 5-year categories was analysed using *t* tests. The dependent variables were Internet access (high access versus low/no access), and the likelihood of using the Internet as a source of each of information and support (likely/very likely compared with unsure/not likely/very unlikely). Those independent variables with a *P* < .25 were included in a backward stepwise logistic regression for each dependent variable. We removed variables until we found an optimal model, based on the Bayesian information criterion. Analyses were conducted in Stata version 11.1 (StatCorp LP, College Station, TX, USA).

## Results

### Sample

We invited 732 eligible survivors to complete and return a survey. Of these, 268 (36.6%) returned a completed survey. The age distribution of responders was significantly different from that of nonresponders, with younger people less likely to return a survey than older people (χ^2^_5_ = 17.2, *P* = .004). Gender, area of residence, type of cancer, and year of diagnosis were not significantly different between responders and nonresponders. As Table 1 shows, participants from a regional location were significantly older and less likely to be employed than those from metropolitan locations. There were no differences between regional and metropolitan participants in terms of cancer type, gender, education, and marital status (see Table 1).

**Table 1:** Sociodemographic characteristics of the sample calculated for those living in a major city or regional area at the time of the survey (n = 268)

		Metropolitan		Regional		Total		Test	*P* value
		n	%	n	%	n	%		
**Age (years) (Mean, SD)**		(57.4, 14.3)		(61.9, 12.0)		(59.5, 13.4)		*F*_1,237_ = 6.74	.01
**Female**		60	40%	51	43%	111	41.4%	χ^2^_1_ = 0.2	.67
**Cancer type**									
	Lymphoma	13	9%	7	6%	20	8%		
	Leukemia	43	29%	29	24%	72	27%		
	Myeloma	25	17%	17	14%	42	16%		
	Non-Hodgkin lymphoma	68	46%	66	56%	134	50.0%	χ^2^_3_ = 2.8	.43
**Education**^a^									
	High school or less	60	40%	50	42%	110	41.0%		
	Vocational training	55	37%	48	40%	103	38.4%		
	University	33	22%	20	17%	53	20%	χ^2^_2_ = 1.2	.55
**Employed**		81	54%	41	34%	122	45.5%	χ^2^_1_ = 11.3	.001
**Married**		108	72.5%	96	81%	204	76.1%	χ^2^_1_ = 1.7	.20
**Total**		149	55.6%	119	44.4%	268			

^a^ Education data were missing for two participants.

### Level of Internet Access

Of the 260 participants who answered the Internet access questions, 204 (78.5%) reported having home Internet access and 67 (26%) reported Internet access at work; 48 (19%) reported having no Internet access and a further 5 (2%) reported no access to the Internet for personal use—that is, 20% were without access to the Internet for personal use. Of those with access (n=207), 167 (80.7%) report daily or weekly use of email.

Table 2 describes the nature of reported Internet access, indicating that approximately 73% of participants reported high levels of Internet access, with regional participants more likely to report connection problems.

**Table 2:** Nature of Internet access for those with access who answered all the access questions (n = 201), and overall level of access for whole sample (n = 254)

Nature of access		Metropolitan (n=111)		Regional (n=90)		Total		χ^2^_2_	*P* value
		n	%	n	%	n	%		
**Frequency of access**									
	Any time	97	87%	74	82%	171	85.1%		
	Most of time	13	12%	13	14%	26	13%	1.92	.38
**Connection problems**									
	None	90	81%	56	62%	146	73%		
	Minor	20	18%	31	34%	51	25%	9.20	.01
**Private**									
	Very	66	60%	66	73%	132	65.7%		
	Moderately	40	36%	19	21%	59	29%	5.34	.07
**Comfortable**									
	Very	84	76%	67	74%	151	75.1%		
	Moderately	27	24%	22	24%	49	24%	1.24	.54
**Can print personal information**									
	Any amount	98	88%	73	81%	171	85.1%		
	Limited amount	6	5%	8	9%	14	7%	2.02	.36
**Confident with Internet**									
	Very	60	54%	42	47%	102	50.8%		
	Moderately	41	37%	29	32%	70	35%	5.90	.05
**Access score^a^**									
	High	106	75.7%	80	70%	186	73.2%		
	Moderate	5	4%	10	9%	15	6%		
	Low	0	0%	0	0%	0	0%		
	None	29	21%	24	21%	53	21%	3.14	.21

^a^ See text for access score calculation. The denominator for access score is the whole sample (ie, includes those with no access).

### Likelihood of Using Various Modes of Information and Support

As Table 3 shows, face-to-face and print were the preferred approaches for receiving both information and support. Approximately 62% of the sample reported they were likely to use the Internet for information, while 27% reported being likely to use the Internet to access support. The main perceived benefits of use of the Internet as a source of either information or support were that it is available anytime (137/253 = 54.2%) and contains a large amount of information (105/253 = 41.5%). A minority of respondents mentioned additional benefits of not needing to travel (81/253 = 32%), low cost (79/253 = 31%), and not requiring personal contact (44/253 = 17%). The perceived disadvantages of Internet-based support were a lack of specificity (102/251 = 40.6%), being too complex (85/251 = 34%), being too impersonal (69/251 = 28%), and difficulty with using the Internet (35/251 = 14%).

**Table 3:** Likelihood (likely/very likely) of using Internet, telephone, print, electronic media, or face-to-face forms of support (n = 251)

Mode		Use for information		Use for support	
		n	% (95% CI^a^)	n	% (95% CI^a^)
Face-to-face		218	87% (83%–91%)	209	83% (79%–88%)
Print		204	81% (76%–86%)	164	65% (59%–71%)
Internet		156	62% (56%–68%)	69	27% (22%–33%)
Electronic		137	55% (48%–61%)	107	43% (36%–49%)
Telephone		131	52% (46%–58%)	96	38% (32%–44%)
**Number of options chosen as likely or very likely**					
	≥2	222	88% (84%–92%)	170	68% (62%–74%)
	1 only	21	8% (5%–12%)	65	26% (20%–31%)
	Print only^b^	4	2% (0%–3%)	11	4% (2%–7%)
	Face-to-face only^b^	13	5 (2%–8%)	52	21% (16%–26%)
	None	8	3 (1%–5%)	16	6% (3%–9%)

^a^ Confidence interval.

^b^ Likely/very likely for item of interest and unsure/not likely/very unlikely to all others.

### Sociodemographic Characteristics Associated With Reported Internet Access and Likelihood of Use

Educational level was significantly associated with reported high Internet access (Fisher exact test *P* < .001) but was not included in the multiple logistic regression model due to a zero cell count (all 53 university-educated participants reported high Internet access). Household size was also associated with high Internet access, with 74% (90/121) of those living with 1 other person and 97% (55/57) of those living with 2 or more people having high access, compared with 66% (21/32) of those living alone (Fisher exact test *P* < .001). This was also not included in the model due to low cell counts. The multiple logistic regression (see Table 4) indicated that younger people were more likely than those who were older to report high Internet access, as were those who were married and those in full- or part-time employment compared respectively with those who were single or not employed. Those who reported that they were likely to use the Internet to find information were more likely to be younger rather than older, to be anxious rather than not anxious, and to have a university degree than were those with only a high school education or vocational training. Participants who were feeling anxious and those in full- or part-time employment, compared with those not employed, were more likely to report being likely to use the Internet as a means of support.

**Table 4:** Logistic regression analysis of factors associated with high reported Internet access, likelihood of using the Internet for information, and likelihood of using the Internet for support

			High or likely	Low or unlikely	Odds ratio (95% CI^a^)	*P* value
**High access (n = 210)**						
	**Age (5 years), mean (SD)**		52.7 (13.2)	65.46 (7.6)	0.89 (0.84–0.94)	<.001
	**Married, n (%)**					
		No	30 (65%)	16 (35%)		
		Yes	136 (82.9%)	28 (17%)	5.63 (2.12–14.94)	.001
	**Employed, n (%)**					
		No	69 (65%)	38 (36%)		
		Yes	97 (94%)	6 (6%)	4.02 (1.37–11.8)	.01
**Likely to use the Internet for information (n = 221)**						
	**Age (5 years), mean (SD)**		52.12 (13.87)	60.68 (10.41)	0.95 (0.93–0.98)	<.001
	**Education, n (%)**					
		High school only	45 (51%)	43 (49%)		
		Vocational training	50 (59%)	35 (41%)	1.3 (0.68–2.46)	.43
		University degree	41 (85%)	7 (15%)	5.06 (1.97–12.98)	.001
	**Anxious, n (%)**					
		No	84 (56%)	66 (44%)		
		Yes	52 (73%)	19 (27%)	2.39 (1.23–4.63)	.01
**Likely to use the Internet for support (n = 221)**						
	**Employed, n (%)**					
		No	22 (20%)	91 (81%)		
		Yes	37 (34%)	71 (66%)	2.53 (1.33–4.81)	.005
	**Anxious, n (%)**					
		No	30 (20%)	120 (80.0%)		
		Yes	29 (41%)	42 (59%)	3.17 (1.66–6.05)	<.001

^a^ Confidence interval.

## Discussion

As approximately three-quarters (73%) of the sample reported a high level of Internet access, such an approach appears to be relatively accessible to most patients. It must, however, be acknowledged that a substantial minority of the sample (20%) reported either having no Internet access at all (18%) or no access for personal use (2%). As higher reported Internet access was associated with higher educational level, younger age, being married, and being employed, those with less access appear to be a potentially isolated and disadvantaged group. Therefore, in order to avoid creating inequity, care should be taken to develop and provide appropriate alternative forms of information and support for such patients. An unexpected finding was that of no reported differences between regional and metropolitan participants regarding access to the Internet, other than greater difficulties with connectivity in regional areas. Therefore, Web-based approaches may indeed assist with improving access to information and support for cancer survivors, across a range of geographic locations. It is likely that adult patients with cancers other than hematologic cancers would similarly benefit from access to Web-based options for information and support.

The reported likelihood of using the Internet for obtaining information or seeking support was relatively low at 62% and 27%, respectively. Studies of Web-based interventions for depression and anxiety found that 78% to 95% of participants took up the offer [21-24]. It may be that the concrete offer of a Web-based program at a time of need is more engaging than the hypothetical possibility proposed in the present study. The samples of patients with a mental illness were younger than the hematologic cancer patient sample and, therefore, likely to be more familiar with Web-based technology.

The data suggest that, while the vast majority of patients reported being likely to use multiple modes for gathering information or seeking support (88% and 68%, respectively), face-to-face and print were the generally preferred forms. Notably, almost 1 in 4 participants reported they would access only one form of support, suggesting that retaining a range of support options is required in order to cater for the support needs of all hematologic cancer patients.

Interestingly, patients’ preference for receiving information via face-to-face or print mode has not changed over time, despite increased accessibility to Internet resources. Previous work by Hinds et al suggested that cancer patients receiving radiotherapy preferred to receive verbal information from their physician in the pretreatment phase and printed information in the posttreatment phase [25]. A more recent systematic review that examined information needs and sources of information across a wider range of cancer patients found that the most frequently cited sources of information were health care professionals and printed materials [26].

In accordance with our findings, one other study has found that cancer patients who were single, older, male, and less educated [15] were less likely than their counterparts to engage with Web-based forms of information or support. Therefore, while Web-based provision of information may be attractive to the majority of patients, those who are less interested in such formats should not be forgotten. The perceived disadvantages of the Internet, particularly complexity and impersonality, also suggest the development of customized Web-based information sources may be useful for patients, rather than relying on generic engine-based searches such as Google. Promotion, careful training, and assistance may reduce some reticence toward newer forms of technology, although print or face-to-face options may need to be retained for those who continue to need or prefer such forms of communication.

An additional new finding is the association between being classified as anxious and a reported likelihood of using the Internet for information and support. This may reflect a greater need or desire for information and support among this group, potentially driving a desire to access available options. Alternatively, anxious cancer survivors may prefer options that require less interpersonal interaction. Other studies support the view that level of anxiety mediates the relationship between seeking information online and using health care services [27].

### Limitations

The low response rate limits the generalizability of the data. However, given the scarcity of data regarding Internet accessibility for cancer patients generally and hematologic cancer patients in particular, these data are the best estimates available. It is possible that a paper-based survey is less likely to be completed by those with a preference for electronic media, resulting in the data providing an underestimate of respondents’ likely use of the Internet as a source of information or support. Low rates of expected use of the Internet, particularly for support, may also be partly due to difficulties in conceptualizing how such support might operate.

### Conclusions

Ensuring that all hematologic cancer patients have equitable access to information and support remains a challenge. While Web-based approaches to information provision appear likely to be accessible and acceptable to the majority of patients, they are less attractive for the provision of support. In addition, more vulnerable patients such as those who are older, single, unemployed, or less educated are particularly likely to require alternative forms of information and support.

## Multimedia Appendix 1

Survey items relating to Internet access and likelihood of use.

## Abbreviations

ARIA+: Accessibility/Remoteness Index of Australia
CI: confidence interval
