# Supplementary material for: Improving Access to Information and Support for Patients With Less Common Cancers: Hematologic Cancer Patients’ Views About Web-Based Approaches
Source: J Med Internet Res. 2011 Dec 21;13(4):e112. doi: 10.2196/jmir.1894 (PMC3278098; doi:10.2196/jmir.1894)
Supplement: Supplementary file 1 [file jmir_v13i4e112_app1.pdf]

Multimedia Appendix 1
Survey items relating to internet access and likelihood of use

**1. Do you have access to the internet?**<br><br>*Please circle ALL that apply*	1. Yes – at home<br>2. Yes – at work<br>3. Yes – at local library or café<br>4. Yes – other (please specify) ___________<br>5. Not at all **→go to Q9**
**2. How often do you use email?**<br><br>*Please circle only ONE answer*	1. Daily<br>2. Weekly<br>3. A few times per month<br>4. Less than monthly<br>5. Not at all
**3. How would you describe your access to the internet for personal use?**<br>*Please circle only ONE answer*	1. Available any time I want to use it<br>2. Available most of the time I want to use it<br>3. Available only some of the time/rarely<br>4. I don't have access to the internet for personal use<br>**→go to Q9**
**4. Do you have any problems with access to the internet for personal use? (eg. loss of connection, low speed, downloading limits)**<br>*Please circle only ONE answer*	1. No problems<br>2. Minor or occasional problems<br>3. Major or frequent problem
**5. How private is the location where you use the internet for personal things?**<br>***(eg. Are you often interrupted? Can others also see what you are looking at?)***<br>*Please circle only ONE answer*	1. Very private<br>2. Moderately private<br>3. Not very private
**6. How comfortable is the location where you usually use the internet for personal things?**<br>*Please circle only ONE answer*	1. Very comfortable<br>2. Moderately comfortable<br>3. Not very comfortable
**7. Are you able to print personal information from the internet?**<br>*Please circle only ONE answer*	1. Yes- I can print as much as I like<br>2. Yes- but I have to limit the amount I print<br>3. No I can't print personal information
**8. How confident are you in using the internet to find information?**<br>*Please circle only ONE answer*	1. Very confident<br>2. Moderately confident<br>3. Not very confident<br>4. I have never used the internet

**9. Often people who have had cancer need information (eg. about cancer treatments, sources of financial help, or help with practical things like transport). If you needed this kind of information, how likely would you be to use the following sources?:**	
**a) Internet information**<br>**(eg. websites that provide treatment information or contact details for support services)**<br>*Please circle only ONE answer*	1. Very likely<br>2. Likely<br>3. Unsure<br>4. Unlikely<br>5. Very unlikely
**b) Telephone information**<br>**(eg. Cancer Council helpline)**<br>*Please circle only ONE answer*	1. Very likely<br>2. Likely<br>3. Unsure<br>4. Unlikely<br>5. Very unlikely
**c) Printed materials**<br>**(eg. books, brochures or magazines)**<br>*Please circle only ONE answer*	1. Very likely<br>2. Likely<br>3. Unsure<br>4. Unlikely<br>5. Very unikely
**d) Electronic media**<br>**(eg. informational DVDs from the Cancer Council, watching relevant TV programs, or listening to the radio)**<br>*Please circle only ONE answer*	1. Very likely<br>2. Likely<br>3. Unsure<br>4. Unlikely<br>5. Very unikely

**e) Face-to-face information**<br>**(eg. talking to health professionals or other patients about managing treatment effects or how to find support services)**<br>*Please circle only ONE answer*	1. Very likely<br>2. Likely<br>3. Unsure<br>4. Unlikely<br>5. Very unlikely

**10. Often people who have had cancer need <u>personal support</u> to cope with feeling down, stressed, anxious, or with trying to stay positive. If you were seeking this kind of help, how likely would you be to use the following types of support?:**	
**a) Internet support**<br>**(eg. online counselling programs, online training in relaxation techniques, or online forums where you can talk to others who have had cancer)**<br>*Please circle only ONE answer*	1. Very likely<br>2. Likely<br>3. Unsure<br>4. Unlikely<br>5. Very unikely
**b) Telephone support**<br>**(eg. the Cancer Helpline, talking to a counsellor on the phone, or being part of a telephone-based support group)**<br>*Please circle only ONE answer*	1. Very likely<br>2. Likely<br>3. Unsure<br>4. Unlikely<br>5. Very unlikely
**c) Printed materials**<br>**(eg. books or brochures from the Cancer Council, or reading information in newspapers or magazines)**<br>*Please circle only ONE answer*	1. Very likely<br>2. Likely<br>3. Unsure<br>4. Unlikely<br>5. Very unlikely
**d) Electronic media**<br>**(eg. Informational DVDs from the Cancer Council, or watching relevant TV programs)**<br>*Please circle only ONE answer*	1. Very likely<br>2. Likely<br>3. Unsure<br>4. Unlikely<br>5. Very unlikely
**e) Face-to-face support**<br>**(eg. visiting your doctor, seeing a psychologist, or attending a face-to-face support group)**<br>*Please circle only ONE answer*	1. Very likely<br>2. Likely<br>3. Unsure<br>4. Unlikely<br>5. Very unikely

**11. Which if any benefits of internet support are important to you?**<br>*Please circle ALL that apply*	1. No personal contact required<br>2. No travel involved<br>3. Available anytime<br>4. Low cost<br>5. Access to a large amount of information<br>6. None of the above<br>7. Other (please specify): ____________________<br>____<br>____
**12. What if any do you think are the main disadvantages of offering support eg. online programs, online discussion forums via the internet?**<br>*Please circle ALL that apply*	1. Too impersonal<br>2. Could be complex or confusing<br>3. May not be specific enough to my situation<br>4. Have trouble using the internet (eg with navigating, reading, or typing)<br>5. None of the above<br>6. Other (please specify): ___________________<br>____<br>____
